# PAPA and FMF in two siblings: possible amplification of clinical presentation? A case report

**DOI:** 10.1186/s13052-019-0705-z

**Published:** 2019-08-23

**Authors:** Maria Cristina Maggio, Isabella Ceccherini, Alice Grossi, Marco Gattorno, Giovanni Corsello

**Affiliations:** 1 Genetic Unit, IRCCS Istituto Giannina Gaslini, Genoa, Italy; 2 Clinica Pediatrica e Reumatologia, Istituto Giannina Gaslini, Genoa, Italy; 3 Department of Health Promotion Sciences Maternal and Infantile Care, Internal Medicine and Medical Specialities “G. D’Alessandro”, University of Palermo, Palermo, Italy

**Keywords:** Familial Mediterranean fever, Pyogenic arthritis, pyoderma gangrenosum and acne syndrome, Colchicine, Canakinumab

## Abstract

**Background:**

Familial Mediterranean Fever is a monogenic autoinflammatory disease, typically characterized by recurrent attacks of fever, serositis, aphthous of oral mucosa, erythema.

“Pyogenic arthritis, pyoderma gangrenosum and acne syndrome” is a rare autoinflammatory disease with variable expression and typically involving joints and skin. Both the diseases are linked by the overproduction of IL-1.

**Case presentation:**

We report on the case of two siblings affected by recurrent attacks of fever, oral aphthous stomatitis, abdominal pain, arthritis, undefined dermatitis at the hands, associated with increased AST, ALT, C-reactive protein, erythrocyte sedimentation rate, serum amyloid A, leucocytosis with neutrophilia. Infectious diseases were excluded. The genetic study for Familial Mediterranean Fever, tumor necrosis factor receptor-associated periodic syndrome, Mevalonate kinase deficiency, showed the homozygous mutation p.M680I of exon 10 in MEFV. Their parents were heterozygous for the same mutation p.M680I, however, the mother showed severe symptoms of FMF (recurrent attacks of fever, arthralgia and arthritis, abdominal pain, thoracic pain), the father showed recurrent pustulosis prevalent on the hands and limbs, with arthralgia and abdominal pain.

Both the patients started colchicine, with an improvement in clinical manifestations and a reduction of serum amyloid A.

For the atypical dermatologic signs present in the two siblings and in the father, the study of other autoinflammatory syndromes was performed with next generation sequencing and showed the heterozygous rare missense mutation of unknown significance: p.(Val408Ile) of PSTPIP1 gene in the two siblings and in the mother, the father was negative.

Canakinumab treatment was started in the younger patient, with the resolution of the clinical symptoms and the normalization of serum amyloid A.

**Conclusions:**

Further studies are needed to better describe the correlation between genotype and phenotype in patients with PAPA syndrome and with PAPA syndrome associated with FMF, considering that the presence of mutations in both genes may amplify clinical presentation and evolution of both diseases.

## Background

Familial Mediterranean Fever (FMF) is a monogenic autoinflammatory disease, secondary to mutations of MEFV gene in the chromosome 16p13, and typically characterized by recurrent self-limiting attacks of fever, arthritis, aphthous changes in lips and/or oral mucosa, erythema, serositis [1, 2]. FMF is caused by dysregulation of the inflammasome, a complex intracellular multiprotein structure, commanding the overproduction of interleukin-1(IL-1). The attacks of FMF can recognize a trigger in infections, stress, menses.

Pyogenic arthritis, pyoderma gangrenosum and acne (PAPA) syndrome is a rare autosomal dominant autoinflammatory disease with incomplete penetrance and variable expression. It involves the proline serine threonine phosphatase-interacting protein 1 (PSTPIP1/CD2BP1) gene on chromosome 15q.

The mutations first described, A230T and E250A, as well as other mutations found on the same gene, increase phosphorylation of PSTPIP1, inducing a higher affinity for the linkage with pyrin than wild type forms [3]. Hence, inflammasome assembly and activation is altered, increasing release of IL-1 beta [4]. The overexpression of IL-1 beta induces an increased secretion of proinflammatory chemokines and cytokines, with the recruitment and activation of neutrophils, managing a neutrophil-mediated inflammatory disease.

This mutation causes the overproduction of the pro-inflammatory cytokine IL-1.

This discovery linked FMF and PAPA syndrome within the same previously described pathway, suggesting a common pathway that could be targeted therapeutically.

The clinical phenotype of PAPA syndrome is characterized by early onset of recurrent episodes of acute aseptic inflammation of the joints, generally occurring in the first two decades of life, followed by typical skin lesions in the third decade, after a clear reduction of the arthritis.

## Case presentation

We report on two siblings, affected by recurrent attacks of fever, oral aphthous stomatitis, abdominal pain, retrosternal and thoracic pain, arthritis, undefined dermatitis at the hands, with palmar maculopapular erythema followed by desquamation. Increased AST, ALT (1.5 x n.v.), C-reactive protein (CRP): 20 x n.v.; erythrocyte sedimentation rate (ESR): > 100, leucocytosis with neutrophilia were present in both patients. Specific IgM and IgG to exclude infection of Epstein-Barr virus [5], Parvovirus, HHV6, Mycoplasma Pneumoniae, pharyngeal swab for *Streptococcus pyogenes* were negative.

Patient 1 is a 16-year-old boy, with recurrent attacks, 3–7 days lasting, of fever, oral aphthous stomatitis, abdominal pain, thoracic pain, arthritis, lumbar pain, palmar maculopapular erythema followed by desquamation, periungual dermatitis with peeling, erythema, acne.

Patient 2 is an 8.4-year-old boy, with recurrent, 3–7 days lasting, attacks of fever, oral aphthous stomatitis, abdominal pain, diarrhoea, vomiting, with − 3 episodes/year of acute abdomen, mimicking acute appendicitis and requiring recovering in surgery emergency unit, retrosternal and thoracic pain, arthritis, dermatitis at the hands, rash at the trunk and at the face, with palmar maculopapular erythema followed by desquamation.

The mother showed recurrent episodes of fever with arthralgia and arthritis, headache, asthenia, abdominal pain, thoracic pain; the father showed recurrent pustulosis prevalent on the hands and limbs, with arthralgia and abdominal pain.

The genetic study for FMF, TNF receptor-associated periodic syndrome (TRAPS), Mevalonate kinase deficiency (MVK) showed the same genetic profile in the two patients. They showed the homozygous mutation p.M680I of exon 10.

Both parents and the 18-year-old sister showed a heterozygous mutation of p.M680I.

The two patients showed increased levels of serum amyloid A (SAA) (> 5–10 x n.v.) far away from recurrent attacks.

Patient 2 started colchicine, with a reduction of the number and length of fever episodes, abdominal pain, arthritis, aphthous stomatitis. However, abdominal pain, arthralgia, vomiting, diarrhoea, dermatitis persisted. SAA levels reduced, still continuing to maintain elevated levels in the free intervals.

For the atypical dermatologic signs present in the two siblings and in the father, the study of other autoinflammatory syndromes was performed using a specific next generation sequencing based gene panel already reported [6]. This allowed to detect a heterozygous rare missense mutation of unknown significance in the two patients and in the mother, the p.(Val408Ile) in the exon 15 of the PSTPIP1 gene.

Patient 1 is continuing colchicine at the dosage of 1,25 mg/day, with the resolution of recurrent attacks of fever, serositis, aphthous stomatitis. However, maculopapular erythema followed by desquamation, periungual dermatitis with peeling and acne persist and he is in follow-up to consider the treatment with canakinumab, a fully human anti-IL-1beta monoclonal antibody will be started in the next months.

Patient 2 started subcutaneous treatment with canakinumab, at the dosage of 2 mg/kg every 4 weeks, maintaining colchicine. After the first 3 doses, fever, abdominal pain, arthralgia persisted even if less severe for intensity and length. The canakinumab dosage was increased at 4 mg/kg/every 4 weeks and the symptoms resolved, with the complete normalization of SAA. However, for the persistence of diarrhoea, colchicine dose will be reduced progressively.

## Discussion and conclusions

The homozygous mutation M680I of MEFV is typically associated with a severe FMF phenotype. The same mutation has been observed also in a remarkable number of heterozygous patients affected with FMF symptoms and FMF-like pictures [7]. This implies that the clinical picture of probands’parents, both carriers of one M680I allele, can be reconducted to their MEFV genotype.

The detection of the rare missense mutation p.(Val408Ile) of the PSTPIP1 gene suggests some considerations. The rarity of the mutation, with an *in-silico* prediction to be damaging though it is still classified as a variant of unknown significance (VUS), in association with a more severe presentation of FMF, has prompted us to take this variant into consideration as a modifier allele.

In our patients, skin lesions were significantly expressed and did not respond to colchicine, however, they were not typical of PAPA Syndrome. In patient 2, canakinumab satisfactory controlled the clinical presentation, with the normalization of SAA. The father showed skin lesions not specific of the PSTPIP1 gene mutation, and he is consistently negative for the presence of any possibly PSTPIP1 causative variant. Other contributory genetic elements able to explain his dermatological manifestations could be investigated in the future by exome study.

In our patients, the association of MEFV and PSTPIP1 mutations, rarely described in the same subject, could represent the amplifying factor of a meaningful clinical picture, expressed as FMF refractory to colchicine treatment, with a more severe clinical presentation, as observed in the two siblings (homozygous however for a significantly severe mutation of exon 10) and in the mother. As the father, she is heterozygous for the same mutation, however, she presents clinical features much more severe, with a poor quality of life.

Significant variation is described in PAPA syndrome presentation from case to case; it is conceivable that the spectrum of the disease is wider than currently seen. Furthermore, the association in the same patient of mutations in the MEFV gene, involving the inflammasome as well, may increase the clinical expression of the disease thus requiring treatment with high dose of anti-IL-1 drugs. Efficacy and safety profile also in paediatric age [8–10] permit to consider canakinumab in low responders to colchicine. Further studies are needed to better describe the correlation between genotype and phenotype in patients with PAPA syndrome and with PAPA syndrome associated with FMF, considering that the presence of mutations in both genes may amplify clinical presentation and evolution of both diseases.

## Data Availability

materials and data of the patient are included in the medical records of the patient.

## Abbreviations

FMF: Familial Mediterranean Fever
CRP: C-reactive protein
ESR: Erythrocyte sedimentation rate
IL-1: Interleukin-1
MVK: Mevalonate kinase deficiency
PAPA syndrome: Pyogenic arthritis, pyoderma gangrenosum and acne
PSTPIP1: Proline serine threonine phosphatase-interacting protein 1
SAA: Serum amyloid A
TRAPS: TNF receptor-associated periodic syndrome
VUS: Variant of unknown significance
